# Cost-effectiveness analysis of Chinese patent medicines for the treatment of postmenopausal osteoporosis in China

**DOI:** 10.3389/fpubh.2025.1596676

**Published:** 2025-09-05

**Authors:** Cong Wang, Xihan Lin, Jinyu Liu, Yu Zhang, Ruxu You

**Affiliations:** ^1^Department of Pharmacy, Union Hospital, Tongji Medical College, Huazhong University of Science and Technology, Wuhan, Hubei, China; ^2^Department of Pharmacy, Tongji Hospital, Tongji Medical College, Huazhong University of Science and Technology, Wuhan, Hubei, China

**Keywords:** osteoporosis, Chinese patent medicines, postmenopausal, cost-effectiveness, Xianling Gubao capsules, Jintiange capsules

## Abstract

**Introduction:**

Evidence indicates that Chinese patent medicines can significantly increase bone mass in patients with osteoporosis and alleviate symptoms associated with low bone density. Although the therapeutic effects of these two drugs have been compared both directly and indirectly, no economic-related studies currently exist. Therefore, this study aims to assess the cost-effectiveness of Xianling Gubao Capsules compared to Jintiange Capsules and non-treatment for postmenopausal osteoporosis from the perspective of Chinese healthcare providers.

**Methods:**

A Markov microsimulation model was employed to estimate the cost-effectiveness of the Xianling Gubao capsule and the Jintiange capsule in a hypothetical cohort of postmenopausal osteoporotic women aged 55 to 74 years with no prior history of fractures, over a treatment period of 6 months. Model parameters, including transition probabilities and costs, were derived from Chinese sources. Efficacy data for the treatments were obtained from two network meta-analyses. Outcomes were expressed as incremental costs per quality-adjusted life-year (QALY) gained. Sensitivity analyses were performed to ensure the robustness of the findings, with a cost-effectiveness threshold established at three times the Gross Domestic Product (GDP) per capita in China ($38,223) per QALY.

**Result:**

Compared to the control group that did not receive drug treatment, the preventive therapy using Chinese patent medicine significantly increased bone mineral density and reduced the probability of fractures across all age groups in the intervention group. The incremental cost-effectiveness ratios (ICERs) for the Jintiange capsule compared to the Xianling Gubao capsule ranged from $11,955 per QALY at age 55 to $9,711 per QALY at age 74, indicating that the cost-effectiveness of the Jintiange capsule improved consistently with age. Sensitivity analyses confirmed the robustness of the results across all parameter variations, with the annual cost of the Jintiange capsule identified as the most sensitive factor.

**Conclusion:**

From the perspective of Chinese healthcare providers, preventive therapy using Chinese patent medicine, when compared to a control group that did not receive drug treatment, resulted in increased bone mineral density and a reduced probability of fractures across all age levels in the intervention group. Additionally, the Jintiange capsule appears to be a cost-effective treatment option for postmenopausal women with osteoporosis.

## Introduction

Osteoporosis is a chronic, progressive skeletal disorder characterized by reduced bone mass, deterioration of bone microstructure, and increased fragility, which collectively elevate the risk of fragility fractures that can severely compromise patients’ quality of life (1). Projections indicate that by 2040, nearly 319 million individuals worldwide will be at risk of osteoporotic fractures, with 55% of these cases expected to occur in Asia (2). According to the national census data from the National Bureau of Statistics of China at the end of 2021, the population aged 60 and above in China was 267.36 million, accounting for 18.9% of the total population. Among this group, the population aged 65 and above was 200.56 million, representing 14.2% (3). The Diagnosis and Treatment Guidelines for Primary Osteoporosis (2022) in China indicate that the prevalence of osteoporosis among women aged 50 and above is 32.1%, which is six times higher than that of men in the same age group (4). Furthermore, the prevalence of osteoporosis significantly increases among females aged 60 and above (1). Osteoporosis can lead to various types of fractures. A study conducted in 2015 estimated that the medical expenses for major osteoporotic fractures in China would reach as high as 11 billion, 20 billion, and 25 billion USD in 2015, 2035, and 2050, respectively (5). Therefore, it is essential to identify safe, effective, and economical treatment options.

Traditional osteoporosis medications include bisphosphonates, parathyroid hormone, selective estrogen receptor modulators, calcium supplements, estrogen replacement therapy, and calcilytics (6–8). While these drugs have demonstrated varying degrees of efficacy in the treatment of osteoporosis, patient compliance remains unsatisfactory due to the occurrence of adverse reactions (9–11). Hormone replacement therapy (HRT), a common treatment for osteoporosis, has been associated with an increased risk of cardiovascular disease and breast cancer (12). Additionally, long-term calcium intake, a routine preventive measure for osteoporosis, has also been linked to a heightened risk of myocardial infarction (13). Given the chronic nature of osteoporosis, it is crucial to balance the associated risks and benefits (14). In China, Chinese patent medicines (CPMs) are ready-made medications formulated in specific dosage forms based on prescriptions or standards guided by the principles of traditional Chinese medicine (15, 16). These medicines are widely utilized in the treatment of osteoporosis (15, 17). Evidence indicates that CPMs can significantly enhance bone mass in patients with osteoporosis and alleviate symptoms associated with low bone mass. The Xianling Gubao Capsule and Jintiange Capsule are two CPMs primarily recommended by various treatment guidelines (1, 18). Although there have been both direct and indirect comparisons of the therapeutic effects of these two medications, no economic evaluations have been conducted (19). Therefore, this study aims to compare the cost-effectiveness of Xianling Gubao Capsules and Jintiange Capsules, as well as non-treatment options for postmenopausal osteoporosis, from the perspective of Chinese healthcare providers, thereby addressing a significant gap in the existing literature.

## Methods

### Study design

This study utilized a 100,000 hypothetical individuals Markov microsimulation model to estimate the cost-effectiveness of Xianling Gubao Capsules and Jintiange Capsules in Chinese postmenopausal women, compared to no intervention. Each cycle lasts 1 year, during which each participant may experience a hip fracture, clinical vertebral fracture, or other types of fractures. Adopting the perspective of Chinese healthcare providers and extending the analysis to a lifetime horizon, the states are continuously updated until the patient’s death. A consistent discount rate of 3% was applied to both costs and health outcomes to account for time preference. The analysis was conducted using TreeAge Pro (Healthcare Version) 2022, in accordance with the Consolidated Health Economic Reporting Standards (CHEERS), as detailed in Supplementary Table 1 (20).

The model meticulously simulated a cohort of Chinese postmenopausal women with no prior history of fragility fractures across various age groups: 55–59, 60–64, 65–69, and 70–74 years. The base case focused on individuals aged 55–59 years. In our model construction and parameterization, age-specific mean and standard deviation (SD) data of BMD shown in Table 1 were derived from Wang et al. (21). The simulation incorporated a normal distribution derived from the mean and standard deviation (SD) values reported by Wang et al., based on the statistical assumption of a large-sample distribution. An initial BMD value was sampled from this normal distribution at the commencement of the Markov microsimulation and subsequently assigned randomly to individual participants. In accordance with established guidelines (1, 18), patients in all groups were administered calcium and activated vitamin D. The cohort was then assigned to receive either Xianling Gubao treatment (Xianling Gubao capsules 1.5 g b.i.d. for 6 months) or Jintiange treatment (Jintiange capsules 1.2 g t.i.d. for 6 months), while the no-intervention group was designated as the status quo.

**Table 1:** Mean BMDs, SDs of the femoral neck in different initial medicated ages of female (g/cm^2^).

Age (years)	Number	Mean	SD	95%CI
55–59	3,152	0.73	0.13	(0.72, 0.73)
60–64	3,155	0.66	0.12	(0.65, 0.66)
65–69	925	0.58	0.11	(0.58, 0.59)
70–74	137	0.55	0.13	(0.53, 0.58)

### Model structure

**Figure 1:**
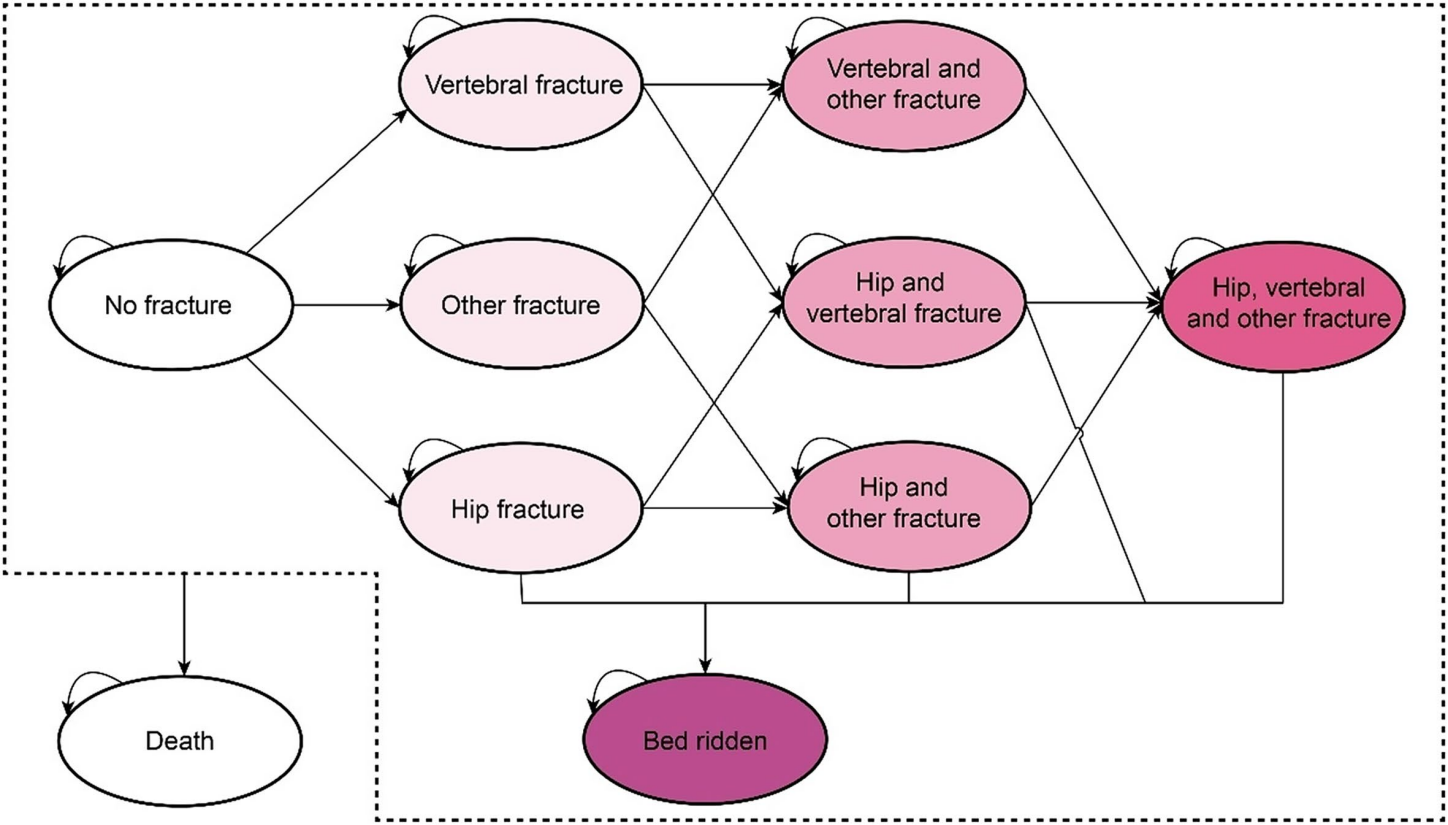
The Markov model structure for the disease progression of postmenopausal women with osteoporosis (omitting the arrow pointing to the death state).

Figure 1 illustrates the structure of the Markov model, which includes the states of no fracture, simple fracture, complex fracture, bedridden due to hip fracture, and death from fracture or other causes. The simple fracture state indicates that individuals had experienced any hip, vertebral, or other types of fractures. The complex fracture state was defined as the occurrence of multiple simple fracture events. It was assumed that a certain proportion of individuals diagnosed with hip fractures may transition to the bedridden state without experiencing any additional fractures.

All patients entered the model in a “no fracture” healthy state. During each cycle, patients could experience a fracture, remain healthy, or die. Patients in the “fracture” state could either remain in the same fracture state if a refracture occurred, transition to another fracture state if a new fracture occurred, or move to the corresponding “post-fracture” state. Among these states, only patients with hip fractures may enter a bedridden state. For instance, patients who have experienced a vertebral fracture might subsequently experience another vertebral fracture or a hip fracture. Fractured patients could not return to the “no fracture” healthy state and would remain in the “post vertebral fracture” or “post hip fracture” state unless another fracture occurs or they entered the bedridden state. Ultimately, all patients were subject to the risk of death, and upon decease, they were transferred to the terminal death state.

### Model parameters

All model parameters were sourced from China whenever possible to ensure their relevance to the local healthcare environment. In the absence of local data, information was synthesized from published literature through systematic literature searches. The input data utilized in the model is presented in the following section (Table 2).

**Table 2:** Estimates of parameters used in the model.

Parameter	Base-case value	Range	Distribution	Source
Relative risk of fracture in individuals with osteoporosis				
RR of hip fractures with complications				
History of previous fractures	1.97	1.12–3.48	Log-normal	(29)
RR of vertebral fractures with complications				
History of previous fractures	1.91	1.50–2.43	Log-normal	(29)
RR of other fractures with complications				
History of previous fractures	1.91	1.50–2.43	Log-normal	(29)
Probability of bedridden after hip fracture	0.136	0.095–0.177	Fixed	(32)
The therapeutic effect of drugs				
Xianling Gubao capsule				(59)
Increase in bone density (g/cm^2^)	0.05	0.01–0.08	Beta	
Treatment time (year)	0.5		Fixed	
Jintiange capsule				(59)
Increase in bone density (g/cm^2^)	0.11	0.03–0.19	Beta	
Treatment time (year)	0.5		Fixed	
Cost (in 2022 China)				
Annual Cost of Medication (US $)				
Xianling Gubao capsule	$93.24	±20%	Triangular	(36)
Jintiange capsule	$574.82	±20%	Triangular	
Annual medical expenses (US $)	$439.78	±20%	Triangular	
Cost of fracture treatment (US$)				
Hip fracture	$7,379.82	±20%	Triangular	(60)
Vertebral fracture	$1,361.12	±20%	Triangular	
Other fracture	$1,758.30	±20%	Triangular	
Annual bedridden care expenses (US$)	$4,948.90	±20%	Triangular	
Health utility (QALY)				
Age-Related Baseline Utility				(41)
55–59	0.88	0.862–0.897	Beta	
60–65	0.869	0.852–0.885	Beta	
66–70	0.827	0.802–0.851	Beta	
71–74	0.808	0.770–0.846	Beta	
Disutility resulting from hip fractures				(42)
First year	×0.776	0.720–0.844	Beta	
subsequent years	×0.855	0.800–0.909	Beta	
Disutility resulting from vertebral fracture				
First year	×0.724	0.667–0.779	Beta	
subsequent years	×0.868	0.827–0.922	Beta	
Disutility resulting from other fracture				(39)
First year	×0.910	0.880–0.940	Beta	
subsequent years	×1			
Utility of Bedridden State	0.192		Fixed	(41)
Discount rate				
Cost	0.03	0–0.05		(35)
QALYs	0.03	0–0.05		(35)

### Transition probabilities

#### Fracture risks

The transition probability of the fracture state was calculated based on age-specific and BMD-specific incidence rates of fragility fractures (Supplementary Table 2). Equations for the incidence of hip, vertebral, and other fractures associated with age and BMD were derived from published epidemiological data on the Chinese population (22–27). The fitted algorithms were evaluated using the R-squared statistic and adjusted for clinical plausibility. The probability of fracture was further modified based on the relative risk associated with a history of previous fractures, as individuals who have experienced any osteoporotic fracture are at an increased risk of subsequent fracture events. These values were obtained from a meta-analysis (28). Additionally, the probability of a bedridden state resulting from a hip fracture was extracted from a prior study conducted in Japan (29).

#### Mortality

Baseline age-specific mortality rates in the general population were extracted by sex and age from the seventh national population census in China (30). Excess mortality rates attributed to hip fractures were derived from published literature on Chinese women by multiplying the age-dependent risk ratio of mortality following a hip fracture (31).

#### Treatment

The efficacy data for Xianling Gubao capsules and Jintiange capsules were derived from a network meta-analysis of 22 randomized controlled trials that compared the effectiveness of various anti-osteoporotic agents (32). This study represents the largest analysis to date concerning the two aforementioned drugs and included a total of 2,016 postmenopausal women diagnosed with primary osteoporosis. Compared to placebo, Xianling Gubao capsules significantly enhanced lumbar and femoral neck BMD (Mean Difference, MD = 0.13, 95% CI [0.03, 0.22], MD = 0.17, 95% CI [0.06, 0.29]). In contrast, Jintiange capsules significantly improved femoral BMD compared to placebo (MD = 0.11, 95% CI [0.03–0.19]).

In addition, treatment-related adverse events were excluded from the model because previous studies did not find any statistically significant differences between patients treated with Xianling Gubao capsules and those treated with Jintiange capsules (33, 34). Therefore, we assumed that treatment-related adverse events had a negligible impact on the costs and outcomes for patients receiving either Xianling Gubao capsules or Jintiange capsules.

#### Health resource use and costs

According to the Chinese Pharmacoeconomic Guidelines (35), the cost evaluation was conducted from the perspective of Chinese healthcare providers. Direct medical costs encompass the expenses associated with treatment regimens resulting from fracture events, direct medical expenses for each health state, and other medical expenditures. The cost data for this section were primarily obtained from a multi-center survey in China (36). The costs of Xianling Gubao capsules and Jintiange capsules were calculated based on the market share of generic drugs and their branded counterparts in China, utilizing official databases from China’s National Medical Products Administration (NMPA) (37). The estimated annual cost of Xianling Gubao capsules was USD 93.24 (3 capsules, twice a day), while the cost for Jintiange capsules was USD 574.82 (3 capsules, three times a day). All related costs were adjusted to 2022 Chinese Yuan (CNY) using the Consumer Price Index (CPI). For reference, the average exchange rate in 2022 was USD 1 = CNY 6.7321.

#### Utilities

Baseline utility data were extracted from published literature to provide reference value for the decision analysis model (38). The utility values were derived from a Chinese large population using EQ-5D. The disutility multiplier associated with post-fracture in the first and subsequent years was derived from meta-analysis (39, 40). Utility for bed-ridden state was collected from a study of Chinese patients provided with nursing care (41).

### Statistical analysis

In base case analysis, using first-order Monte Carlo simulation, total costs and QALYs for each treatment with Xianling Gubao capsules, Jintiange capsules, and no treatment were estimated at different starting ages of 55, 60, 65, and 70 years. To estimate the cost-effectiveness of Xianling Gubao capsules and Jintiange capsules, an incremental cost-effectiveness ratio (ICER) was calculated by dividing an incremental cost by an incremental quality-adjusted life-year (QALY) to obtain the cost per QALY gained. To explore key drivers of parameters, deterministic sensitivity analyses were conducted, and parameters assessed and their ranges are shown in Table 2. Probabilistic sensitivity analyses were conducted by a second-order Monte Carlo simulation with 1,000 iterations and selecting the assigned parameters distributed randomly (shown in Table 2). Following the analyses, cost-effectiveness acceptability curves were illustrated to determine the probability of being cost-effective for each strategy based on an assumed willingness-to-pay (WTP) threshold of three-time GDP per capita in China ($38,223) per QALY gained.

## Results

### Model validation

**Figure 2:**
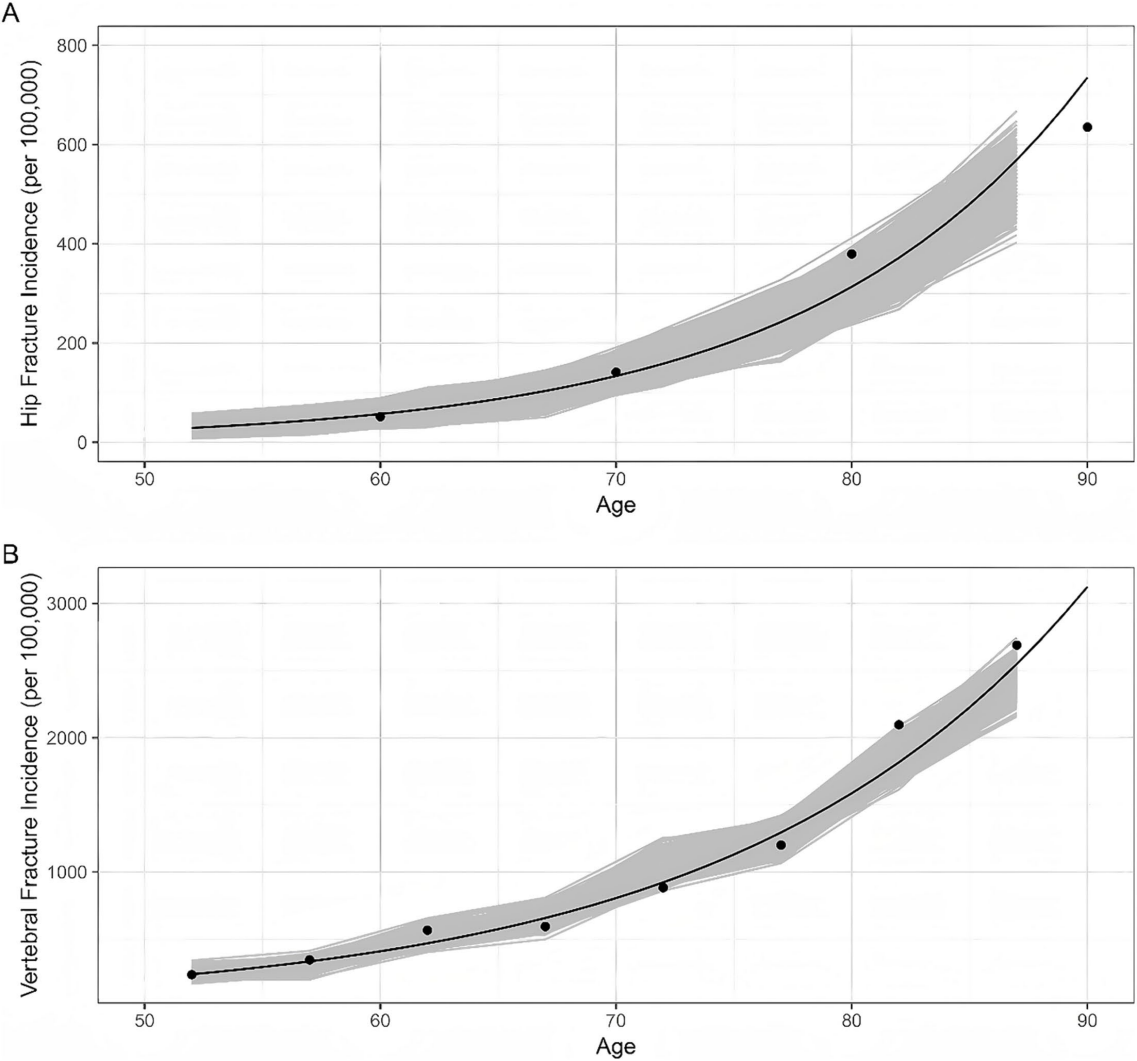
Model validation results. Dots represent the data reported by epidemiological surveys in China. Lines represent the trend curves fitted by the data above-mentioned. Shaded areas represent the model outputs of 1,000 simulations. **(A)** Hip fracture incidence; **(B)** Vertebral fracture incidence.

This model was validated by comparing the age-specific incidence of hip fractures and clinical vertebral fractures per year with estimates from published epidemiological surveys (shown in Figure 2) (23, 42).

### Base-case analyses

Table 3 presents the costs, QALYs, and ICERs for Xianling Gubao or Jintiange compared with no treatment at the starting ages of 55, 60, 65, and 70 years. Compared with the control group without drug treatment, the preventive treatment with Chinese patent medicine increased bone mineral density and reduced fracture probability at all age levels in the intervention group. Model simulation results based on females aged 55–59 showed that the use of Xianling Gubao capsules reduced hip fracture incidence by 8.20% and vertebral fracture incidence by 12.03%, with an increase in total per capita cost of $119.21 and an increase in quality-adjusted life years (QALYs) by 0.0379. The use of Jintiane capsule reduced hip fracture rates by 18.33% and vertebral fracture rates by 30.05%, with an increase in total per capita cost of $956.38 and an increase in QALYs by 0.08. Under the willingness-to-pay (WTP) threshold of three times China’s per capita GDP, the use of traditional Chinese medicine for preventive treatment had a cost-effective advantage. Moreover, compared with the Xianling Gubao capsule, the Jintiange capsule was cost-effective (ICER: $11,955/QALY).

**Table 3:** Cost-effectiveness of Xianling Gubao compared with Jintiange or no treatment at the starting ages of 55, 60, 65, and 70 years.

Treatment Strategy	Probability of patients experiencing a fracture within the study period		Cost, $	QALYs	ICER, $/QALY
	Hip fracture	Vertebral fracture			
55–59 years					
No Treatment	6.71%	21.03%	7174.94	13.0513	Ref
Xianling Gubao Treatment	6.16%	18.50%	7294.16	13.0892	3,147
Jintiange Treatment	5.48%	15.47%	8131.32	13.1313	11,955
60–64 years					
No Treatment	7.30%	23.58%	6791.35	11.4976	Ref
Xianling Gubao Treatment	6.90%	20.81%	6883.21	11.5373	2,313
Jintiange Treatment	5.94%	17.40%	7681.96	11.5879	9,858
65–69 years					
No Treatment	6.60%	20.40%	6075.22	9.9942	Ref
Xianling Gubao Treatment	6.09%	18.04%	6184.11	10.052	1886
Jintiange Treatment	5.29%	14.91%	6978.64	10.0784	10,731
70–74 years					
No Treatment	7.46%	23.00%	5572.26	8.0271	Ref
Xianling Gubao Treatment	6.49%	20.63%	5646.1	8.0875	1,221
Jintiange Treatment	5.86%	17.16%	6393.42	8.1116	9,711

For the population aged 60–74, the use of traditional Chinese medicine for preventive treatment still had a cost-effective advantage. Compared with the Xianling Gubao capsule, the Jintiange capsule was cost-effective (ICER: $9858-10731/QALY) under the condition of WTP being 3 times China’s per capita GDP.

### One-way sensitivity analyses

**Figure 3:**
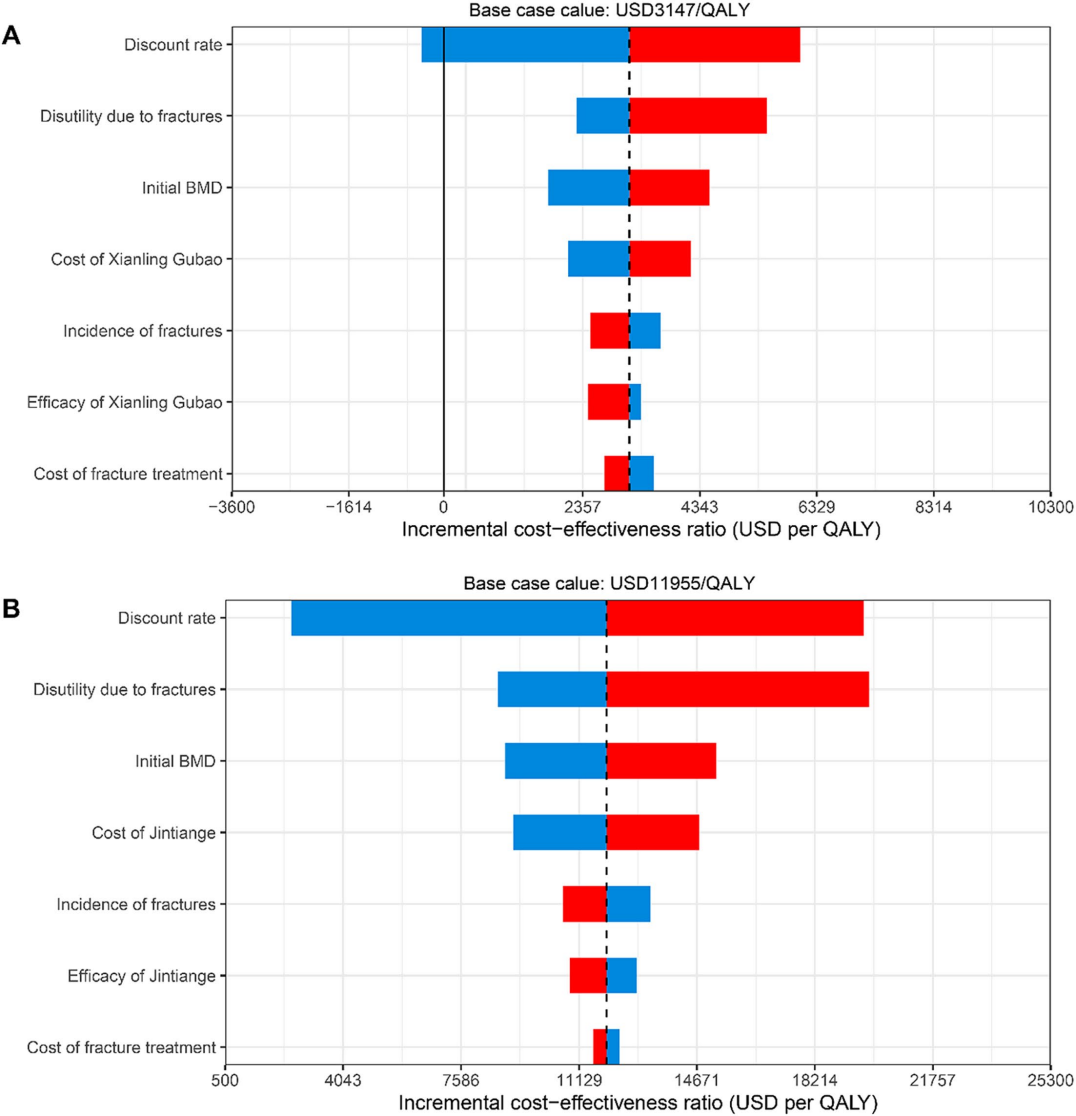
Tornado diagrams for one-way sensitivity analyses on the incremental cost-effectiveness ratio of Xianling Gubao capsule compared with Jintiange capsule or no treatment. Blue and red represent the ICER results in lower limit and upper limit values of parameters used, respectively. **(A)** Xianling Gubao; **(B)** Jintiang.

One-way sensitivity analyses comparing the Xianling Gubao capsule with the Jintiange capsule or no treatment indicated that the ICERs were most sensitive to the discount rate, the loss of utility due to fractures, the first-year BMD of the study population, and drug acquisition costs (Figure 3). The graph demonstrates that the cost-effectiveness results of CPM treatment are relatively stable and unaffected by changes in these parameters. It is cost-effective to initiate preventive treatment with CPMs for individuals with below-average BMD who experience a significant impact on their quality of life due to fractures.

### Probabilistic sensitivity analyses

**Figure 4:**
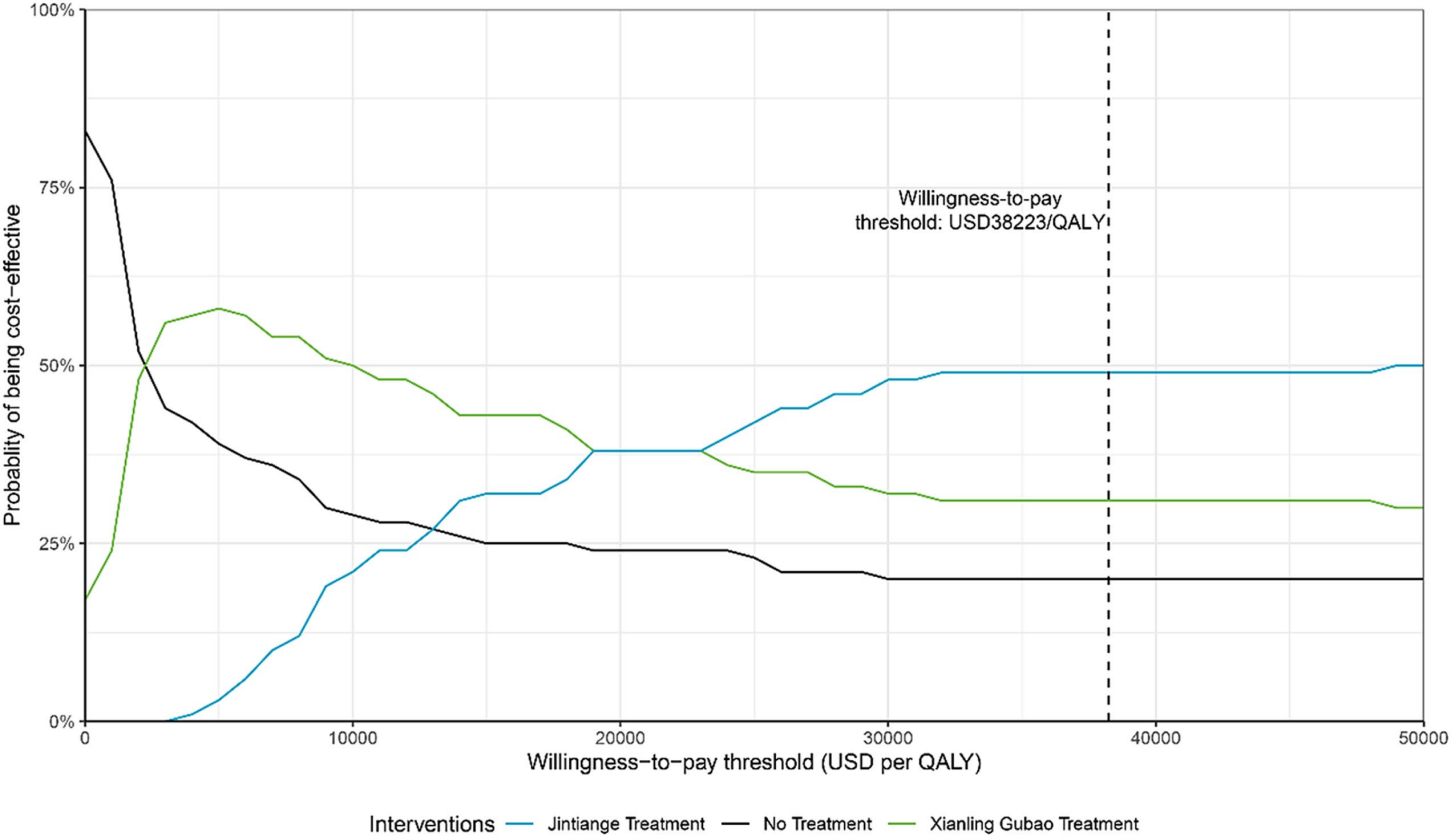
Cost-effectiveness acceptability curve of Xianling Gubao capsules compared with Jintiange capsules or no treatment.

At a WTP threshold of $3,000 (approximately 0.24 times GDP per capita), the use of CPMs for preventive treatment in the female population aged 55–59 was deemed cost-effective. When the WTP exceeded $24,000 (about 1.88 times per capita GDP), the economic benefits of using the Jintiange capsule became more pronounced compared to the Xianling Gubao capsule. Under the WTP conditions established in the study, the probabilities of the Xianling Gubao capsule and the Jintiange capsule being cost-effective were 31 and 49%, respectively (Figure 4).

## Discussion

Previous systematic reviews had indicated a relative scarcity of cost-effectiveness research concerning CPMs (43). In this study, we constructed a Markov model based on life status to simulate the long-term effects of CPMs on the treatment outcomes of menopausal women with osteoporosis. Our base analysis suggests that, at a WTP threshold of $3,000 per QALY, the use of CPMs for preventive treatment in postmenopausal women aged 55 to 59 was cost-effective. Furthermore, at a WTP threshold equivalent to three times China’s per capita GDP, the combined use of Jintiange capsules was cost-effective across all age groups. This study provided reference values for future long-term economic evaluations of CPMs for osteoporosis in postmenopausal women.

The primary treatment approaches for bone loss diseases such as osteoporosis are HRT and bisphosphonates. Continuous HRT was associated with a high risk of breast cancer and endometrial cancer, as well as coronary artery issues and other cardiac disorders, while bisphosphonates could lead to osteonecrosis of the jawbone and skeletal system (44–46). Due to these adverse effects, the clinical use of HRT and bisphosphonates is limited. Therefore, new treatment strategies were needed to develop osteoporosis treatments that are less likely to cause adverse reactions to some extent (47). CPMs had gained popularity due to their minimal adverse effects while effectively treating various ailments. Traditional Chinese Medicine (TCM) had been utilized to address a range of orthopedic conditions, particularly osteoporosis, fractures, and rheumatism, with notable success (48, 49). Xianling Gubao and Jintiange treatments played significant roles in managing osteoporosis with CPMs, making them the preferred choice for treating postmenopausal osteoporosis according to clinical application guidelines for Chinese patent medicines. Xianling Gubao capsules consist entirely of CPMs (50), which help regulate the balance of serum calcium and phosphorus deposition, enhance levels of vitamin D3 and alkaline phosphatase (ALP), and improve bone mineral density through the synergistic effects of various natural herbs targeting multiple pathways (51, 52). The primary component of Jintiange capsules was artificial tiger bone powder. The physical and chemical properties, as well as the pharmacological effects were consistent. Researchers studying the serum of patients with osteoporosis found that Jintiange capsules inhibited the κB inhibitor signaling pathway by downregulating the overexpression of osteopontin, ultimately reducing MMP-3 expression. This action may strengthen the kidneys and bones, alleviate inflammation and pain, and combat osteoporosis (53). Furthermore, recent studies have identified Jintiange capsules as the first CPM that could effectively improve primary osteoporosis and enhance muscle strength (54). While the therapeutic potential of CPMs in managing osteoporosis is evident, there is a lack of economic studies to support their widespread adoption. Therefore, further economic evaluations are necessary to inform clinical and policy decisions regarding the integration of CPMs into standard osteoporosis treatment protocols.

The choice of research perspective in economic evaluation determines the measurement range of cost. Our results demonstrated that the use of CPMs preventive treatment appears to be a cost-effective treatment option for postmenopausal osteoporotic women at the starting age of 55 from the perspective of Chinese healthcare providers. Our results also revealed that the cost-effectiveness of Jintiange Treatment improved with an increase in the starting age. Our findings were consistent with previous economic evaluations in which the Jintiange treatment was generally cost-effective compared with the Xianling Gubao treatment (19, 32, 55). We did not find any studies that were different from this conclusion, which may be caused by the lack of relevant economic studies. However, pharmacoeconomic assessments were based on data from clinical trials or real-world data, and further CPMs clinical trials and real-world studies were recommended.

Currently, the pharmacoeconomic studies on the treatment of osteoporosis in menopausal women published both at home and abroad mainly concentrate on RANKL inhibitors, bisphosphonates, and other Western medicine preparations. There have been relatively few studies on CPMs for treating osteoporosis in menopausal women. Only Lai Fuchong et al. (34) carried out an economic analysis of Xianling Gubao capsules and Jintiange capsules in the treatment of type-I osteoporosis. The results indicated that the cost-effectiveness ratio of alendronate combined with Xianling Gubao capsule was the lowest, followed by alendronate combined with Jintiange capsule. Nevertheless, the limitations of this study include the lack of ICERs, which are essential for definitively determining the most cost-effective treatment strategy. Furthermore, the data were gathered from a restricted patient sample across two hospitals, without taking into account potential confounding factors. This may have an impact on the generalizability of the results. The current study aims to fill this gap by presenting a comparative economic assessment of Xianling Gubao Capsules and Jintiange Capsules in postmenopausal women of various age groups at the start of treatment. Such an analysis is crucial for promoting the rational clinical use of CPMs in osteoporosis management, providing valuable perspectives for healthcare providers and policymakers when considering the incorporation of these treatments into standard care guidelines.

Although there were studies on the synergistic effect of CPM combined with conventional Western medicine in the treatment of osteoporosis (56), unfortunately, we had not found any direct comparative studies on the treatment of osteoporosis in postmenopausal women with CPM and Western medicine, including safety, efficacy and economy. Although no direct research evidence comparing CPM and Western medicine in the treatment of osteoporosis in postmenopausal women has been found so far, our team had fully realized the importance of this research gap for clinical practice and health policy-making. Currently, we were actively promoting the preparatory work for subsequent observational and real-world studies to provide solid methodological support for future research design. We were confident that through standardized research implementation and rigorous data analysis, we will gradually fill this research gap and provide more comprehensive evidence-based basis for the optimization of treatment decisions for osteoporosis in postmenopausal women.

As with any modeling research, our analyses had certain limitations. First, due to the absence of epidemiological data fully consistent with the study population, the efficacy parameter of the model transition probability involved a series of assumptions and corrections. This might deviate to some extent from the actual disease outcomes. Second, owing to the lack of relevant research, our data on the efficacy of drug treatment were solely obtained from a network meta-analysis. Although this was the largest study related to both drugs, the data dated back to 2016, and the study endpoint was bone mineral density (BMD), which was used to predict the incidence of fractures subsequently. This could introduce bias to our results. Third, to maintain model parsimony, we did not incorporate adverse events. However, serious adverse events caused by Xianling Gubao capsules and Jin Tiange in the treatment of osteoporosis are considered rare (33, 34). Thus, they were unlikely to affect the results of our cost-effectiveness analyses. Similarly, this was a commonly adopted assumption in previous economic evaluations (57, 58). The impact of treatment-related adverse events on costs and outcomes can be regarded as minimal. Nevertheless, if data on adverse events become available, they should be integrated into the model.

## Conclusion

From the perspective of the Chinese healthcare providers, compared with the control group without drug therapy, the preventive treatment with Chinese patent medicine increased bone mineral density and reduced fracture probability at all age levels in the intervention group, and Jintiange capsule appears to be a cost-effective treatment choice for postmenopausal osteoporotic women. This study provides valuable information to both clinical practitioners and decision-makers in ensuring the rational use of Chinese patent medicine, especially in the face of the growing clinical and economic burden of osteoporotic fractures in China.

## Data Availability

The original contributions presented in the study are included in the article/Supplementary material, further inquiries can be directed to the corresponding author.
