# Supplementary material for: Cost-effectiveness analysis of Chinese patent medicines for the treatment of postmenopausal osteoporosis in China
Source: Front Public Health. 2025 Sep 5;13:1596676. doi: 10.3389/fpubh.2025.1596676 (PMC12446330; doi:10.3389/fpubh.2025.1596676)
Supplement: Supplementary file 2 [file Table_2.DOCX]

| **Equation** | **Base case** | **Range** | **Reference** |
| --- | --- | --- | --- |
| Equations for age- and BMD- dependent incidence rate of fracture |  |  |  |
| Hip fracture | $\frac{3.3889276976\cdot{10}^{-6}\cdot exp(0.085358\cdot Age)}{1.058239611}\cdot{1.4}^{-z}$ | ±30% | [1-4] |
| Vertebral fracture without previous vertebral fracture | $\frac{6.990593537\cdot{10}^{-5}\cdot exp(0.067803\cdot Age)}{1.208314829}\cdot{1.85}^{-z}$ | ±30% | [1-3] |
| Vertebral fracture with previous vertebral fracture | $\frac{5.64811735\cdot{10}^{-3}\cdot exp(0.033355\cdot Age)}{1.208314829}\cdot{1.85}^{-z}$ |  | [1-3] |
| Other fracture | $\frac{1.638631537\cdot{10}^{-5}\cdot exp(0.084641\cdot Age)}{1.058239611}\cdot{1.4}^{-z}$ | ±30% | [2,3,5,6] |

**Supplemental table 2. Equations for estimating probabilities of osteoporotic fracture.**

BMD: bone mineral density, Z: Z - score = (current BMD - average BMD for a given age) ÷ SD of BMD

**References**

1. Clynes, M.A.; Harvey, N.C.; Curtis, E.M.; Fuggle, N.R.; Dennison, E.M.; Cooper, C. The epidemiology of osteoporosis. *British medical bulletin* **2020**, *133*, 105-117, doi:10.1093/bmb/ldaa005.

2. Malle, O.; Borgstroem, F.; Fahrleitner-Pammer, A.; Svedbom, A.; Dimai, S.V.; Dimai, H.P. Mind the gap: Incidence of osteoporosis treatment after an osteoporotic fracture - results of the Austrian branch of the International Costs and Utilities Related to Osteoporotic Fractures Study (ICUROS). *Bone* **2021**, *142*, 115071, doi:10.1016/j.bone.2019.115071.

3. Zhang, C.G.; Feng, J.N.; Wang, S.F.; Gao, P.; Xu, L.; Zhu, J.X.; Jial, J.L.; Liu, L.L.; Liu, G.Z.; Wang, J.X.; et al. Incidence of and trends in hip fracture among adults in urban China: A nationwide retrospective cohort study. *PLoS medicine* **2020**, *17*, doi:ARTN e100318010.1371/journal.pmed.1003180.

4. Lau, E.M.; Woo, J.; Leung, P.C.; Swaminthan, R. Low bone mineral density, grip strength and skinfold thickness are important risk factors for hip fracture in Hong Kong Chinese. *Osteoporosis international : a journal established as result of cooperation between the European Foundation for Osteoporosis and the National Osteoporosis Foundation of the USA* **1993**, *3*, 66-70, doi:10.1007/BF01623375.

5. Bow, C.H.; Cheung, E.; Cheung, C.L.; Xiao, S.M.; Loong, C.; Soong, C.; Tan, K.C.; Luckey, M.M.; Cauley, J.A.; Fujiwara, S.; et al. Ethnic difference of clinical vertebral fracture risk. *Osteoporosis international : a journal established as result of cooperation between the European Foundation for Osteoporosis and the National Osteoporosis Foundation of the USA* **2012**, *23*, 879-885, doi:10.1007/s00198-011-1627-9.

6. Tsang, S.W.; Kung, A.W.; Kanis, J.A.; Johansson, H.; Oden, A. Ten-year fracture probability in Hong Kong Southern Chinese according to age and BMD femoral neck T-scores. *Osteoporosis international : a journal established as result of cooperation between the European Foundation for Osteoporosis and the National Osteoporosis Foundation of the USA* **2009**, *20*, 1939-1945, doi:10.1007/s00198-009-0906-1.
